# Insight into the bacterial gut microbiome of the North American moose (*Alces alces*)

**DOI:** 10.1186/1471-2180-12-212

**Published:** 2012-09-19

**Authors:** Suzanne L Ishaq, André-Denis G Wright

**Affiliations:** 1 Department of Animal Science, College of Agriculture and Life Sciences, University of Vermont, 203 Terrill Building, 570 Main Street, Burlington, VT, 05405, USA; 2 Department of Medicine, University of Vermont, 111 Colchester Ave, Burlington, Vermont, 05401, USA

**Keywords:** Colon, Gut microbiome, Rumen, Vermont, 16S rRNA

## Abstract

**Background:**

The work presented here provides the first intensive insight into the bacterial populations in the digestive tract of the North American moose (*Alces alces*). Eight free-range moose on natural pasture were sampled, producing eight rumen samples and six colon samples. Second generation (G2) PhyloChips were used to determine the presence of hundreds of operational taxonomic units (OTUs), representing multiple closely related species/strains (>97% identity), found in the rumen and colon of the moose.

**Results:**

A total of 789 unique OTUs were used for analysis, which passed the fluorescence and the positive fraction thresholds. There were 73 OTUs, representing 21 bacterial families, which were found exclusively in the rumen samples: Lachnospiraceae, Prevotellaceae and several unclassified families, whereas there were 71 OTUs, representing 22 bacterial families, which were found exclusively in the colon samples: Clostridiaceae, Enterobacteriaceae and several unclassified families. Overall, there were 164 OTUs that were found in 100% of the samples. The Firmicutes were the most dominant bacteria phylum in both the rumen and the colon. Microarray data available at ArrayExpress, accession number E-MEXP-3721.

**Conclusions:**

Using PhyloTrac and UniFrac computer software, samples clustered into two distinct groups: rumen and colon, confirming that the rumen and colon are distinct environments. There was an apparent correlation of age to cluster, which will be validated by a larger sample size in future studies, but there were no detectable trends based upon gender.

## Background

North American moose, (*Alces alces*), are the largest browsing ruminant of the deer family Cervidae, and preferably inhabit young hardwood forests, deciduous mixed forests, and salt rich wetland habitats that have an abundance of woody browse and salty aquatic vegetation [1-4]. In northern latitudes, such as Vermont, moose have traditionally done well, although unregulated hunting and deforested habitats caused a severe decline in the Vermont population during the 20^th^ century [5]. It was not until 1993 that moose hunting became regulated again in Vermont and remains strictly controlled by the state. Vermont provides a wide variety of habitats, with one of the most suitable regions being in the northeastern corner of the state. Known as the Northeast Kingdom, the area is rich in bogs and swamps, and is comprised of over 75% deciduous or mixed forests with growth of various maturities [6]. This area also supports the highest concentration of moose in the state [6] and traditionally has the highest hunter success rates: ranging from 38-70% from 2006 to 2009 [7,8], making it an excellent site for sample collection.

Like all ruminants, moose have a specialized digestive system with a four chambered stomach that allows a complex consortium of symbiotic microorganisms to ferment plant matter that the animal cannot breakdown on its own, especially cellulose [9,10]. During the process of fermentation, hydrogen, ammonia, carbon dioxide, and methane gas are produced [11], as well as volatile fatty acids (VFAs) such as acetate, butyrate, and propionate. These VFAs are released into the rumen where they can be absorbed and used by the ruminant as a source of energy [11-13].

Limited work has previously been done using classical microbiology to identify organisms found in the rumen of moose [14]. One male moose from Alaska was shot in August of 1985, and bacteria which were isolated and characterized consisted of *Streptococcus bovis* (21 strains), *Butyrivibrio fibrisolvens* (9 strains), *Lachnospira multiparus* (7 strains), and *Selenomonas ruminantium* (2 strains) [14].

For the present study, the second generation (G2) PhyloChip (PhyloTech Inc., California) was used to survey rumen and colon samples for the presence and presumptive identification of bacteria. The G2 PhyloChip uses 16S rRNA gene sequences to rapidly type bacteria and methanogens in a mixed microbial sample without the use of cloning or sequencing [15,16]. The PhyloChip contains approximately 500,000 probes on its surface, representing over 8,400 species of bacteria and roughly 300 species of archaea [17]. There are 11, 25mer, probes that are designed to hybridize to each specific taxon, allowing for specificity in determining taxa present [17]. Depending on what the probes are designed to target, the PhyloChip can be used to differentiate between different serotypes of *Escherichia coli*, or determine the presence of a species regardless of strain. It is already a popular bacterial screening method for air [15], water [18], and soil [19,20], and has recently gained favor for digestive tract samples [21,22]. Due to their specificity and sensitivity, DNA microarrays have also been used to categorize diseased and healthy states [22,23].

The major objectives of the present study were to type the bacteria present in rumen and colonic samples, and to compare these findings with other studies of ruminants and herbivores. Given that moose are large browsing herbivores [3], it was hypothesized that the bacterial populations in the browse-fed wild moose would be more closely related to bacterial populations found in other browse/forage fed animals. This study reports on the bacteria found in the rumen and colon of the North American moose, as well as how these environments relate to other studies of the gut microbiome in various species.

## Results

### Quantitative Real-Time PCR

Mean bacteria cell densities were calculated for each rumen sample using standard curves generated by Bio-Rad’s CFX96 software. Based on a regression line created using the bacterial standards (R^2^ = 0.997), estimated cell density ranged from 8.46 × 10^11^ to 2.77 × 10^12^ copies of 16S rRNA/g in the rumen (Table 1).

**Table 1:** Estimated densities (16S rRNA copy numbers per gram wet weight) of bacteria in the rumen (R) of the moose in October, 2010, Vermont

**Sample**	**Bacterial copies of 16S rRNA/g (SEM)**
1R	8.46 x 10^11^
2R	1.61 x 10^12^
3R	2.57 x 10^12^
4R	2.02 x 10^12^
5R	9.36 x 10^11^
6R	1.21 x 10^12^
7R	2.77 x 10^12^
8R	1.34 x 10^12^
Mean (SEM)	1.66 x 10^12^ (7.27 x 10^11^)

All figures based on calculations using standard curves generated by the Bio-Rad CFX manager program: bacteria (R^2^ = 0.997).

### PhyloChip array

#### Combined rumen and colon

**Figure 1:**
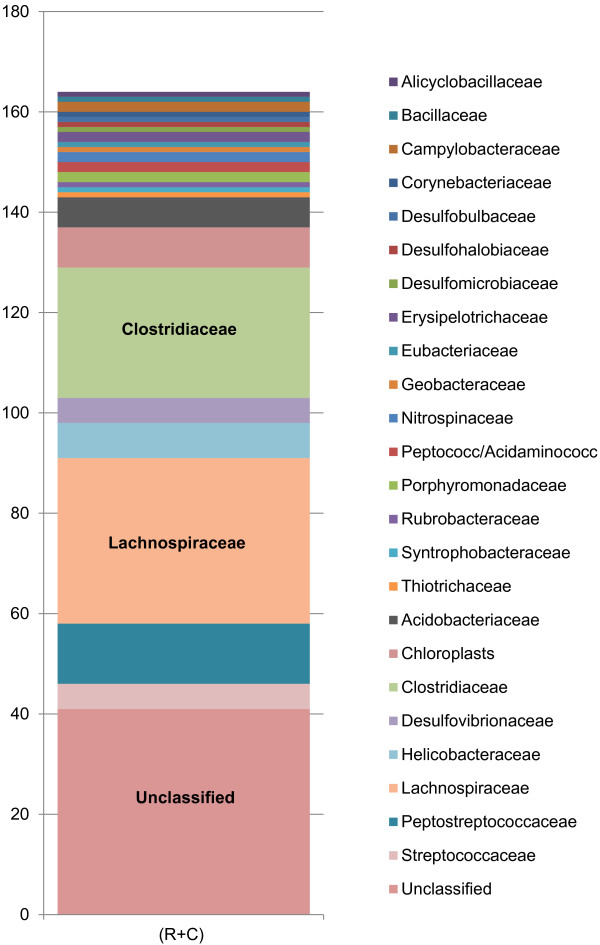
**The OTUs found common in all samples (rumen and colon).** 164 OTUs found common to all samples (n = 14). The Unclassified sections are broken down by phyla in Figure 2a.

**Figure 2:**
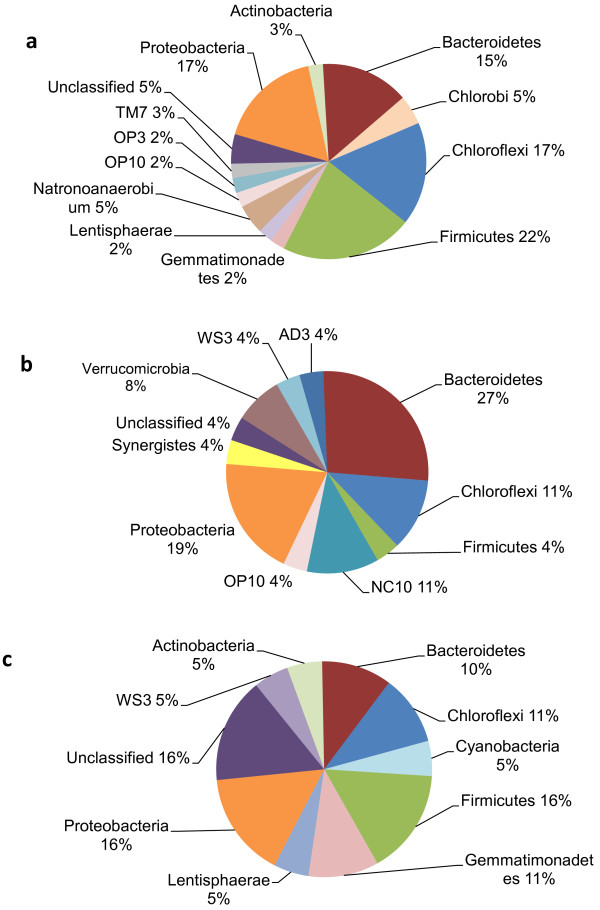
**Breakdown of unclassified families by phylum.** (**a**) OTUs present in all 14 samples. There were 41 OTUs found exclusively in the rumen that were not classified down to the family level. (**b**) OTUs found exclusively in the rumen. There were 22 OTUs found exclusively in the rumen that were not classified down to the family level. (**c**) OTUs found exclusively in the colon. There were 19 OTUs found exclusively in the colon that were not classified down to the family level. Several are candidate phyla and are named by where they were discovered: AD3, soil in Virginia and Deleware, USA; OP3 and OP10, now Armatimonadetes, Obsidian Pool hot spring in Yellowstone National Park, USA; NC10, Null Arbor Caves, Australia; TM7, a peat bog in Gifhorn, Germany; WS3, a contaminated aquifer on Wurtsmith Air Force Base in Michigan, USA.

A total of 789 unique OTUs were used for analysis which passed the fluorescence and the positive fraction thresholds. Total numbers for each taxonomic group found are listed for each sample (Table 2), which represent raw data before initial screening. There were 789 total distinct OTUs that were found in all the samples combined; 267 Firmicutes, 225 Proteobacteria, and 72 Bacteroidetes being the major phyla. Not all OTUs were found in every sample, but out the total 789 OTUs there were 164 OTUs, comprising 25 bacterial families, which were found across all 14 samples (Figure 1). The most abundant of these families were unclassified, 25%; Lachnospiraceae, 20%; Clostridiaceae, 16% and Peptostreptococcaceae, 7%. The remaining 21 families represented less than 4% each of the OTUs found in all 14 samples (Figure 1). The OTUs with unclassified families were then classified by phyla; of the 25% of OTUs with unclassified families, the phyla Firmicutes represented 22%, Proteobacteria and Chloroflexi were 17% each, Bacteroidetes was 15%, and all others represented 5% or less (Figure 2a).

**Table 2:** Total number of taxa found in each sample, before screening for analysis but after background noise was removed and including only OTUs with > 0.92 positive fraction

**Sample**	**Phylum**	**Class**	**Order**	**Family**	**Sub-family**	**OTU**
1R	20	42	59	83	94	367
2R	21	43	63	90	103	395
3R	19	38	51	75	83	308
4R	23	44	58	80	94	374
5R	23	46	67	97	109	465
6R	23	43	56	84	97	382
7R	22	43	57	86	100	379
8R	23	45	69	98	116	432
**Mean rumen**	**22**	**43**	**60**	**87**	**100**	**350**
1C	16	33	45	63	72	331
2C	18	36	54	78	90	378
3C	15	30	40	54	65	307
6C	17	34	50	72	84	374
7C	26	49	82	124	146	597
8C	21	42	66	98	115	488
**Mean colon**	**19**	**37**	**51**	**82**	**95**	**413**

Not all OTUs were found in every sample.

Many of the unclassified sequences were presumptively identified in PhyloTrac, as well as in GenBank, based upon the environment where they were found as most of them are uncultured, thereby providing an interesting, if subjective, means of comparison ( Additional file 1: Table S1 and Additional file 2: Tables S2). Unclassified sequences in the moose were related to a range of environmental sequences including 102 “termite gut clone” OTUs, 20 “rumen clone” OTUs, 20 “forest soil/wetland clone” OTUs, 16 “swine intestine/fecal clone” OTUs, six “human colonic clone” OTUs, six “sludge clone” OTUs, four “penguin dropping clone” OTUs, four “chicken gut clone” OTUs, two “human mouth clone” OTUs and a large number of “soil clone” and “water clone” OTUs from various environments. While many of the forest soil/wetland, soil and water clones may represent transient populations that are picked up from the environment, these data correlate with summer diets of moose in Vermont, namely woody browse in forested areas and aquatic plants found in bogs and marshes.

#### Rumen samples

**Figure 3:**
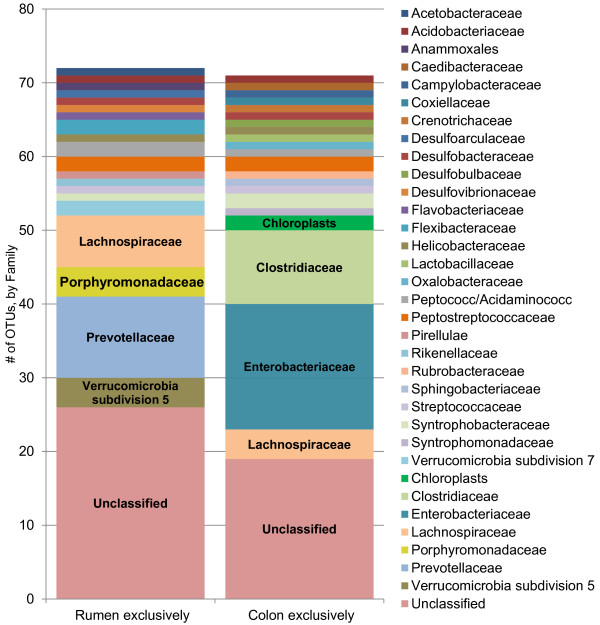
**A comparison of the OTUs exclusive to the rumen or the colon.** A comparison of the 73 OTUs exclusive in the rumen (n = 8) or 71 OTUs exclusive in the colon (n = 6), by family. Families with three or more associated OTUs are labeled in the chart; all other families with two or fewer OTUs are labeled via the legend. The Unclassified sections are broken down by phyla in Figure 2b, and 2c, respectively.

The rumen samples contained 575 total OTUs; 192 Firmicutes, 142 Proteobacteria, and 66 Bacteroidetes being the dominant phyla. In the rumen samples, there was a range of 308 to 465 OTUs/sample, and an average of 350 OTUs/sample (Table 2). There were 237 OTUs found across all eight rumen samples and, of these, 73 OTUs were exclusive to the rumen, representing 21 families (Figure 3). The OTUs with unclassified families were assigned by phyla (Figure 2b), with the dominant phyla being Bacteroidetes, 27%; Proteobacteria, 19%; and Chloroflexi and NC10 with 11% each. NC10 is a candidate phylum consisting of uncultivated and uncharacterized bacteria that is currently named after the location where the bacteria were sampled, Nullarbor Caves, Australia. All other phyla represented 10% or less of OTUs with unclassified families (Figure 2b). Of the unclassified sequences found exclusively in the rumen, there were 51 termite gut clones, 36 marine, wetland, or waterway sediment clones, 13 fecal or colon clones, 11 rumen clones, nine soil clones, and seven sludge clones.

A previous study on rumen microorganisms in the moose [14] identified *Streptococcus bovis* (21 strains), *Butyrivibrio fibrisolvens* (9 strains), *Lachnospira multiparus* (7 strains), and *Selenomonas ruminantium* (2 strains). The present study found *Streptococcus bovis* strains ATCC 43143 and B315 in every sample except for 1C and 2R. *Butyrivibrio fibrisolvens* and *B. fibrisolvens* strain LP1265 were found in all samples except for 3R, 6R, 2C and 3C, whereas *Butyrivibrio fibrisolvens* strain WV1 was found in 8C only. *Lachnospira multiparus* was not present on the chip. However, all 14 samples did contain *Lachnospira pectinoschiza*, as well as *Selenomonas ruminantium* strains S20 and JCM6582.

#### Colon samples

The colon samples contained a total of 658 OTUs; 248 Firmicutes, 194 Proteobacteria and 46 Bacteroidetes. The colon samples ranged from 307 to 597 OTUs/sample, with an average of 413 OTUs/sample (Table 2). There were 235 OTUs that were found across all six colon samples, and of these, 71 OTUs were exclusive to the colon, representing 22 families (Figure 3). Again, the OTUs with unclassified families were assigned by phyla (Figure 2c), with the dominant phyla being Firmicutes, Proteobacteria and Unclassified, 16% each; Gemmatimonadetes and Chloroflexi, 11% each, and Bacteroidetes, 10%. All other phyla represented 10% or less of OTUs with unclassified families (Figure 2c). Again, many unidentified sequences were listed as uncultured clones by location found. The unidentified sequences found exclusively in the colon were related to52 “termite gut clone” OTUs, 20 “marine, wetland, or waterway sediment clone” OTUs, 10 “soil clone” OTUs, eight “fecal/colon clone” OTUs, eight “sludge clone” OTUs and five “rumen clone” OTUs.

### UniFrac analysis

**Figure 4:**
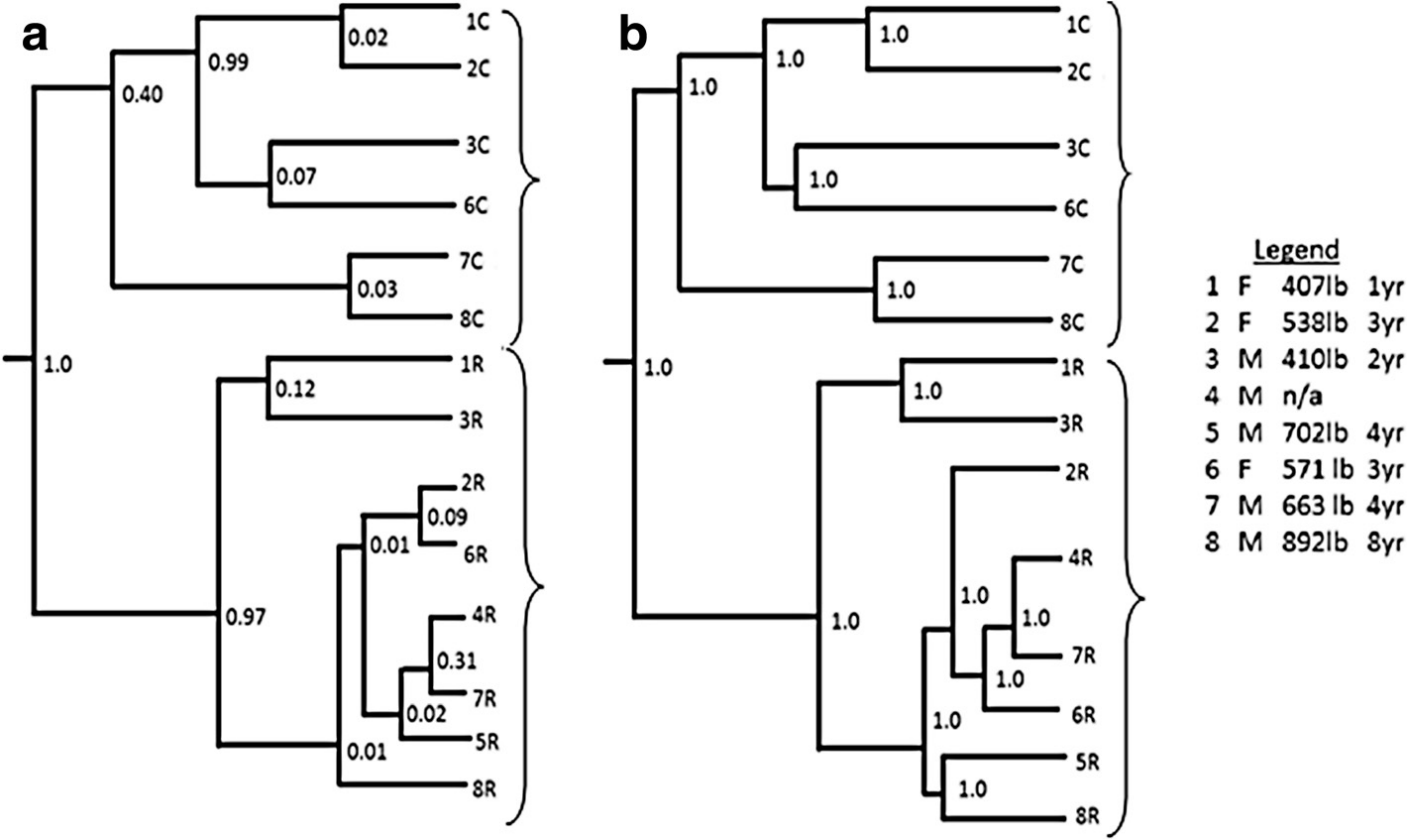
**Jackknife environment clustering in UniFrac, by sample.** (**a**) An unweighted UniFrac algorithm and (**b**) a weighted UniFrac algorithm were used, and were not normalized as different evolutionary rates of gene did not need to be accounted for. Jackknife counts for each are provided for each node. The weighted UniFrac algorithm takes into account abundance of sequences, and is better suited to analysis of mixed bacterial samples. Samples are labeled by individual moose (1–8) and sample type (rumen, R or colon, C), and gender, weight and age information is provided in the legend.

**Figure 5:**
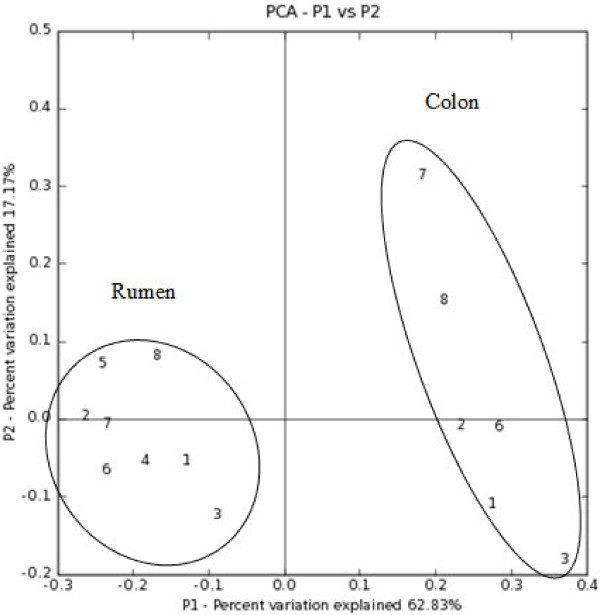
**Principal component analysis (PCA) scatterplot of the environments using the weighted UniFrac algorithm.** Samples are labeled by number (1–8), and groups are shown.

P-test significance was run using all 14 samples together and 100 permutations, resulting in a corrected p-value of < 0.01, designating that each sample was significantly different from each other. Environment clusters and jackknife values are provided (Figure 4), showing a statistical measurement of the correctness of the tree created. The weighted algorithm accounted for the relative abundance of sequences in a sample, which is typical for environmental samples. UniFrac and PhyloTrac both clustered the rumen and colon samples into two distinct groups: the first node was present 100% of the time in the unweighted and weighted UniFrac clusters. The branching pattern for the rumen group is different between UniFrac algorithm (Figure 4) and between programs (Figure 5). However, the branching pattern for the colon group is identical between PhyloTrac, and the unweighted and weighted UniFrac outputs. A principal component analysis (PCA) scatterplot (Figure 5) was also created using the weighted algorithm, which grouped the rumen and colon samples separately.

The rumen samples also tentatively clustered by age/weight in the unweighted UniFrac output (Figure 4a), with the youngest/lightest two grouped together (185 kg., 1-yr old; 186.36 kg, 2-yrs old), the two 3-yr old females, grouped together (244.55 and 259.55 kg), and the three oldest/heaviest males (301.36 kg, 4-yrs old; 319.09 kg, 4-yrs old; and 405.45 kg, 8-yrs old) grouped together with a male of unspecified age/weight. The age/weight clusters within the rumen in the weighted UniFrac output (Figure 4b) were not the same as with the unweighted output, nevertheless, some clusters remained (c.f. Figure 4a and 4b).

## Discussion

The major objective of this study was to identify bacteria present in the rumen and colon content samples of the North American moose. This is the first time that the rumen and colon bacterial populations of the moose have been evaluated on a large scale (i.e. PhyloChip), with the last work published in 1986 [14]. While Dehority’s [14] results give the present study an indication of the bacterial population within the rumen of moose, the findings were limited by a sample size of one animal and the constraints of classical microbiology. Anaerobic gut microorganisms are difficult to culture, which continues to present a major obstacle in gut microbial identification. However, genetic analysis, such as microarray and high-throughput sequencing, allow microbes to be studied before they are grown in a pure culture.

One drawback of using the PhyloChip, and indeed with all methods that forego culturing, is the inability to distinguish between live and dead microbes. It also cannot distinguish between colonizing versus transient species, such as the green sulfur bacteria in the phylum Chlorobi or green non-sulfur bacteria of Chloroflexi, both of which are photosynthetic and picked up by the moose during feeding. Careful analysis of the data is required to properly interpret the results. However, even dead and transient bacterial populations can have a profound impact on the resident bacteria as well as the host, whether by releasing harmful components when lysed, such as Lipid A, or providing DNA which may be taken up by live cells in the rumen, as in plasmids that contain genes that confer antibiotic resistance. Is important to take a holistic view to prevent marginalizing potentially important species. Like all methods that rely on PCR amplification, PhyloChip is also subject to PCR bias. This is mediated during sample preparation by running multiple reactions per sample and minimizing the number of cycles.

Rumen samples were consistently clustered separately from the colon samples by PhyloTrac and UniFrac and there were 174 OTUs that were exclusive to either the rumen or the colon; confirming that the rumen and the colon are two distinct environments. Similar findings were reported in a study using fecal samples from sheep [24], as a non-invasive means of modeling the rumen bacteria from captive exotic animals where it is impractical to obtain rumen contents. It was concluded that bacterial concentrations and species in the colon were not reliably predictive of the bacterial concentrations or species in the rumen [24].

The rumen contained an average of 1.66 × 10^12^ copies of 16S rRNA/g (± 7.27 × 10^11^ SEM). This is comparable to other ruminants: 5.17 × 10^11^ cells/g (± 3.49 × 10^11^) for Norwegian reindeer [25], 1.86 × 10^11^ cells/g (± 9.68 × 10^10^) and 5.38 x 10^11^ cells/g (± 2.62 x 10^11^) for Svalbard reindeer [26] in April and October, respectively, and 1.60 × 10^11^ cells/g (± 1.35 x 10^11^) for Canadian dairy cattle [27].

The dominant phylum in the moose rumen was Firmicutes with 192 OTUs, followed by Proteobacteria with 142 OTUs and Bacteroidetes with 66 OTUs. Firmicutes is often the dominant phylum in gut microbiomes, and many of those found in the moose were of the class Clostridia, containing sulfate-reducing bacteria (SRB), which can be pathogenic, endospore forming, and found in soil. Sundset et al. [28] reported that in rumen samples taken from reindeer in Svalbard, the bacteria cultivated were mainly from the class Clostridia. It was noted that *Fibrobacter succinogenes*, *Ruminococcus albus*, and *R. flavefaciens* were not found in the rumen of the reindeer [28], although this may simply be a bias of the cultivation approach. *Fibrobacter* and *Ruminococcus* are both cellulolytic and have previously been found in the rumen of reindeer [25,29]. However, in the present study, *F. succinogenes* and *R. albus* were not found, despite both species being present on the chip with multiple strains. *Ruminococcus flavefaciens* was detected in several samples, but only a few of its 11 probes matched, making the result insignificant. *Ruminococcus obeum* was detected in the present study.

In a recent paper studying rumen bacteria in dairy cattle, Firmicutes was the dominant phylum in four cattle rumen samples when using full length 16S rRNA clone libraries, but was only dominant in three samples with Proteobacteria being dominant in one sample when using partial 16S rRNA clone libraries or environmental gene tags [30]. Gamma- and alpha-Proteobacteria have been shown to be type I and type II methanotrophs, respectively, meaning they utilize methane as their source of carbon. In the present study, the species *Enterobacter cloacae*, of the class *g*amma-Proteobacteria, was found in the moose, and in a non-lactating Holstein cow based on PCR of the 16S rRNA gene to target methanotrophs [31].

In a comparison between the moose rumen data and a study using the PhyloChip and samples from the crop of the wild folivorous bird, the hoatzin [21], similarities arise. Godoy-Vitorino et al. [17] showed that bacteria from the crop of the hoatzin clustered into distinct groups by age: chicks (n = 3), juveniles (n = 3) and adults (n = 3). This correlates with the present study, as the rumen samples clustered by age/weight in the unweighted, and to some extent, in the weighted UniFrac jackknife clustering. As in the moose, some of the differential families found in the crop of the adult hoatzin included Lachnospiraceae, Acidobacteriaceae, Peptostreptococcaceae, Helicobacteraceae and Unclassified (phyla: Proteobacteria, Cyanobacteria, NC10, Chloroflexi, etc.) [17]. The total number of taxonomic groups discovered for hoatzin chicks, juveniles and adults ranged from 37–40 phyla, 47–49 classes, 88–90 orders, 147–152 families, 305–313 subfamilies, and 1351 to 1521 OTUs, an increase over moose, which possibly arises from grouping three samples onto one chip, as was done with the hoatzin samples [21].

In the study by Godoy-Vitorino et al. [21], as well as the current study, OTU cutoff level was predetermined by the PhyloTrac program (i.e. <97%). However, Godoy-Vitorino et al. [17] used a pf = 0.90 to determine if an OTU was present, meaning that 90% of the probes for that OTU were positive. When a pf value of 0.90 was applied to the current study, effectively lowering the number of probes that needed to be positive to be a match for that OTU, the average number of OTUs present rose from 350 to 488 for the rumen and from 413 to 524 for the colon. This suggests that moose either have only a relatively few bacterial species in large quantities, or that there is a wide variety of bacteria found in the moose which are unique and unable to hybridize to the probes found on the G2 PhyloChip. The PhyloChip has recently been shown to overestimate species diversity [32]. The major drawback to using DNA microarray chips is that only known sequences can be used as probes, thus rendering the chips ineffective for discovering and typing new species [33]. The G2 PhyloChip was created in 2006, thus any new taxa that have been identified since then will not be present on the chip, and any re-classification of sequences that are currently on the chip can only be noted by using the most current version of PhyloTrac. These data will be validated and expanded upon using high-throughput DNA sequencing and cultures.

Despite the many similarities between bacteria found in the rumen of the moose to the hoatzin, reindeer and the previous moose study, there are many bacterial families found in the present study which were not mentioned in any of the previous studies. However, many of these bacterial families have been noted in the foregut of the dromedary camel, a pseudo-ruminant with a three chambered stomach. In a recent study by Samsudin et al. [34], the following bacterial families were found in the foregut dromedary camels (n = 12) as well as the rumen of the moose in the present study (though not in every rumen sample): Eubacteriaceae, Clostridiaceae, Prevotellaceae, Lachnospiraceae, Rikenellaceae, Flexibacteraceae, Bacteroidaceae, Erysipelotrichaceae, Bacillaceae, Peptococcoceae, and Peptostreptococcaceae. Wild dromedary camels in Australia survive on a high fiber forage diet [34], which is closer to the diet of wild North American moose. This may explain why the bacterial populations in wild camels appear to be closer to moose than that of wild reindeer, which eat a diet rich in lichens, despite the reindeer and the moose being members of the Cervidae family.

In the rumen, there were 51 sequences found that were listed as being related to termite gut clones, yet many more similarities can be found between the moose and the termite gut, which have compartmentalized guts containing microbes. *Treponema primitia* strain ZAS-1, as well as five other *Treponema* species, were found in the moose rumen in the present study, and 109 *Treponema* phylotypes and species were previously found in the termite gut [35]. *Treponema primitia*, belonging to the phylum Spirochetes, is an acetogenic microorganism capable of degrading mono- and disaccharides such as cellulose or xylan [35]. Bacteroidetes, Chlorobi, Cyanobacteria, Firmicutes and Proteobacteria clones were also discovered in the termite [35], as well 49 phylotypes which represented three new candidate orders in the phylum Fibrobacteres.

To our knowledge, no studies exist using PhyloChip analysis on the fecal samples of herbivores. However, many other colon studies exist, focusing on medically significant pathogens in humans. In a recent study on irritable bowel syndrome, the bacterial families in healthy rats were Rhizobiaceae, Peptococcaceae/Acidaminocoocus, Clostridiaceae, Lachnospiraceae, Intrasporangiaceae, Succinivibrionaceae, Alteromonadaceae, Paenibacillaceae and Flavobacteriaceae [36]. Of these, only Peptococcaceae/Acidaminocoocus, Clostridiaceae and Lachnospiraceae were found in the moose. In a separate study, fecal samples from cervid species in Norway were tested for colon bacteria that were known pathogens to humans using selective culturing techniques [37]. In that study, *E. coli* O103 was found in 41% of the samples, *E. coli* O26 and O145 were found in small amounts, and *E. coli* O111 and O157 were not found at all [37]. In addition, no cervid fecal samples were positive for *Salmonella*, although one roe deer (*Capreolus capreolus*) sample was positive for *Campylobacter jejuni jejuni*[37]. In the present study, several samples contained *Salmonella, E. coli*, or *Campylobacter* species, although no strains of verocytotoxic (e.g. O157:H7) or uropathogenic (e.g. CFT073) *E. coli, Shigella* or *Campylobacter jejuni jejuni* were found. However, all of the moose colon samples contained *Citrobacter freundii*, a nitrate reducing bacteria commonly found in the environment, which is known to be an opportunistic pathogen in humans.

The moose colon contained 658 OTUs, of which 248 were Firmicutes and 46 Bacteroidetes. In a 2006 study of the mouse gut microbiome in lean and *ob/ob* obese mice, it was discovered that transfaunation with microorganisms from the obese mouse intestine into the lean mice caused increased weight gain and fat deposition [38]. It is important to note that the bacteria in the obese mice had significantly higher proportions of Firmicutes than Bacteroidetes [38].

## Conclusions

The work presented here provides the first insight into the bacterial populations in the digestive tract of the North American moose. While the G2 PhyloChip is an excellent tool for identifying known bacteria, it contains only 300 archaeal sequences, which were not utilized because bacterial-specific primers were used. Furthermore, there is currently no microarray that is designed to identify protozoa or fungi. Next generation (high-throughput) sequencing is needed to validate the bacterial population findings of the present study, as well as identify the protozoal, archaeal and fungal populations present in the moose rumen. The PhyloChip, like all methods that do not rely on culturing, cannot be used to differentiate between transient and colonizing species. It can be assumed that some species found in the moose are simply passing through the digestive tract, having been picked up from the environment, and are not colonizing the tract. Despite this, these transient bacteria may still have an impact on the dynamics within the rumen, and it is important to take a holistic approach when looking at mixed environmental samples. It is also possible that some of these unclassified bacteria which are presumed transient, such as the soil or water clones, are actually colonizing the moose digestive tract and are simply unique to moose.

## Methods

### Sample collection

All samples were obtained with permission of licensed hunters through the Vermont Department of Fish and Wildlife. Whole rumen (R) and colon (C) contents were collected from moose shot during the October 2010 moose hunting season in Vermont. Samples were collected by hunters within 2 h, if not sooner, of death and put on ice immediately. Hunters were given a written set of instructions about sample collection, and had been instructed verbally as well, to fill the collection containers with material taken from well inside the rumen and colon, and to seal the container quickly to minimize overexposure to oxygen. Samples were then transferred to the laboratory within 24 h, and stored at −20°C until DNA extraction. A total of eight rumen and six colon samples (Table 3) were collected from eight moose. Twelve of the samples were paired rumen and colon contents from the same animal, and two rumen samples did not have corresponding colon samples. Moose were weighed and aged, by examining the wear and replacement of the premolars and molars of the lower jar, by Vermont Fish and Wildlife biologists at the mandatory reporting stations.

**Table 3:** Statistics for samples taken from moose shot in October 2010 in Vermont during the moose hunting season

**Moose**	**Sample location**	**Sample name**	**Gender**	**Weight, dressed carcass (kg)**	**Approx. age (yr)**
1	Rumen	1R	F	185	1
	Colon	1C			
2	Rumen	2R	F	244.55	3
	Colon	2C			
3	Rumen	3R	M	186.36	2
	Colon	3C			
4	Rumen	4R	M	N/A	N/A
5	Rumen	5R	M	319.09	4
6	Rumen	6R	F	259.55	3
	Colon	6C			
7	Rumen	7R	M	301.36	4
	Colon	7C			
8	Rumen	8R	M	405.45	8
	Colon	8C			

### DNA extraction

Samples were fully thawed, and 0.25 gram aliquots of either rumen content or colonic material, were used for extraction. DNA was extracted from all 14 samples using the repeated bead-beating plus column (RBB + C) method [39], and the QIAamp DNA Stool Mini Kit (QIAGEN, Germantown, Marlyand). DNA was quantified using a NanoDrop 2000C Spectrophotmeter (ThermoScientific, California), and the purity of the DNA extract was verified using gel electrophoresis to molecular weight. DNA extract was also PCR amplified to test quality and verified using gel electrophoresis to determine correct PCR amplicon length prior to quantitative real-time PCR, or hybridization to the PhyloChip.

### Quantitative Real-Time PCR

Real-time PCR was used to calculate bacterial concentrations in each sample, and was performed using a CFX96 thermocycler (Bio-Rad, Hercules, CA), using universal bacterial primers 1114-F (5’-CGGCAACGAGCGCAACCC-3’) and 1275-R (5’-CCATTGTAGCACGTGTGTAGCC-3’) [40]. Each reaction contained 12.5μL of the iQ SYBR Green Supermix kit (Bio-Rad, Hercules, CA): 2.5 μl of each primer (40 mM), 6.5μL of ddH_2_o, and 1μL of the initial DNA extract which was diluted to approximately 10 ng/μL. The external standard for bacteria, as previously described [40], was a mix of *Ruminococcus flavefaciens* and *Fibrobacter succinogenes* that were serially diluted over four logs.The protocol consisted of an initial denaturing at 95°C for 15 min, then 40 cycles of 95°C for 30s, 60°C for 30s, 72° for 1 min. This was followed by a melt curve, with a temperature increase 0.5°C every 10s from 65°C up to 95°C to check for contamination. Data were analyzed using the CFX Manager Software v1.6 (Bio-Rad, Hercules, CA).

### PhyloChip

DNA (25–50 ng/μl) was sent to the University of Vermont’s Microarray Core Facility for genotyping using the G2 PhyloChip (PhyloTech Inc., San Francisco, CA). There, the 16S rRNA gene of bacteria was PCR amplified using the universal bacterial primers 27 F (5’-AGAGTTTGATCCTGGCTCAG-3’) and 1492R (5’-CTACGGCTACCTTGTTACGA-3’) [41], quantified, fragmented, labeled with biotin, and hybridized according to manufacturer’s proprietary instructions. Each amplified sample was hybridized to its own chip, creating 14 total data sets. The analysis platform used was an Affymetrix 7 G scanner, and Gene Chip Operating System (GCOS). Data generated is available online at ArrayExpress, accession number E-MEXP-3721.

### Analysis

PhyloChip data were analyzed using the software program PhyloTrac v2.0 (available from http://www.phylotrac.org). PhyloTrac automatically removed background noise as the average of the two least intense fluorescence signals in each chip quadrant, and used internal standards to create a linear scale to normalize fluorescence intensity with concentration of that sequence in the original sample [17]. The 16S rRNA sequences on the chip were grouped into Operational Taxonomic Units (OTUs) based on a 97% or greater sequence identity, which was predetermined by the program. For each OTU, there are 11 perfect-match probes, and 11 mismatch probes, which are always analyzed in pairs. For an OTU to be considered a positive match to a probe, the signal intensity must be 1.3X the intensity of the mismatch probe [13]. The positive fraction is a measure of how many perfect-match probes matched out of the total number of probe pairs for that OTU. For this study, a positive fraction of 0.92 was used to determine the presence of an OTU in a sample; for each OTU, 92% of the perfect-match probes were positive. A mean intensity threshold of 100 was used, so that only OTUs with signal intensity greater than that were included in the analysis. All 14 sample files were used in the comparison.

**Figure 6:**
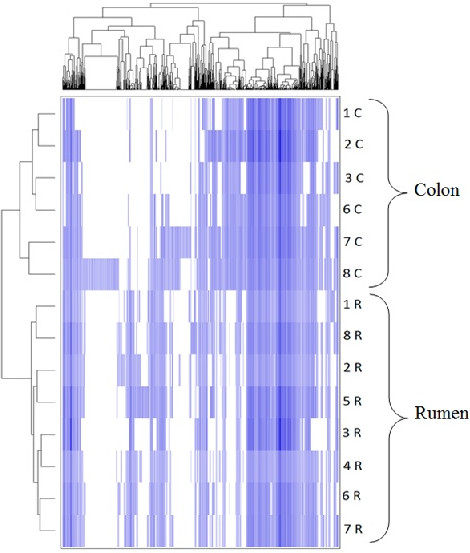
**Distribution of PhyloChip OTU’s for all 14 samples.** Samples (rumen and colon) are arranged in rows and are clustered on the vertical axis (y-axis). OTU’s are arranged vertically and are on the horizontal axis (x-axis). Clustering was done for each using Phylotrac’s heatmap option with Pearson correlations and complete linkage algorithms.

Data were evaluated down to the taxonomic level of family for most analyses since each OTU represented more than one species [32]. A heatmap (Figure 6) showing the presence or absence, and relative intensity of each OTU was created using all 14 samples. Samples were arranged in rows and were clustered on the vertical axis. OTUs were arranged vertically and were clustered on the horizontal axis. Clustering was done using Phylotrac’s heatmap option with Pearson correlation, a measure of the correlation between two variables, and complete linkage algorithms (farthest neighbor), which clusters based on the maximum distance between two variables.

UniFrac (available from http:// bmf2.colorado.edu/unifrac/), an online statistical program, was used to analyze PhyloChip data [42,43] and to confirm the clustering functions of PhyloTrac. Data were exported from PhyloTrac for analysis using the UniFrac statistical software. P-test significance was run using all 14 environments together and 100 permutations, to determine whether each sample was significantly different from each other. A p-value of < 0.05 states that the environments were significantly clustered together. Two Jackknife environment clusters were performed using 100 permutations, the weighted and unweighted UniFrac algorithms, and 307 minimum sequences to keep (UniFrac default for the specified conditions). Jackknife counts were provided for each node, representing the number of times out of 100 that a node was present on the tree when the tree was repeatedly rebuilt. A Jackknife percentage of >50% is considered significant. A principal component analysis (PCA) scatterplot was also created using the weighted algorithm, a chart which arranged two potentially related variables into unrelated variables on a graph, revealing underlying variance within the data.

## Competing interests

The authors declare that they have no competing interests.

## Authors’ contributions

SI carried out all DNA extraction, PCR, PhlyoTac and Unifrac analysis, and drafted the manuscript. AW conceived of the study and participated in its design, and edited the manuscript. Both authors approved the final manuscript.

## Supplementary Material

Additional file 1 **Table S1.** Genus/Identifier and GenBank # of sequences in selected families, found in all rumen samples (n = 8), sequences are non-exclusive to the rumen. Click here for file

Additional file 2 **Table S2.** Genus/Identifier and GenBank # of sequences in selected families, found in all colon samples (n = 6), sequences are non-exclusive to the colon. Click here for file
