# Supplementary material for: Insight into the bacterial gut microbiome of the North American moose (Alces alces)
Source: BMC Microbiol. 2012 Sep 19;12:212. doi: 10.1186/1471-2180-12-212 (PMC3585231; doi:10.1186/1471-2180-12-212)
Supplement: Additional file 1 — Table S1. Genus/Identifier and GenBank # of sequences in selected families, found in all rumen samples (n = 8), sequences are non-exclusive to the rumen. [file 1471-2180-12-212-S1.docx]

**Supplementary Table 1** Genus/Identifier and GenBank # of sequences in selected families, found in all rumen samples (n=8), sequences are non-exclusive to the rumen.

| **Rumen** | |
| --- | --- |
| **Genus or Identifier** | **GenBank ASCN #** |
| **Clostridiaceae** | |
| *Clostridium* | X71852.1, AB093546.1 |
| cow rumen clone | AY244908.1 |
| Great Artesian Basin clone | AF407695.1 |
| Forested wetland clone | AF523923 |
| *Lachnospiraceae* | AF550610.1 |
| Oral clone | AF481208.1 |
| sludge clone | AF482434.1 |
| Swine intestine clone | AF371790.1, AF371796.1 |
| Termite gut clone | AB100478.1, AB100483.1, AB100493.1, AB100479.1,  AB100476.1, AB100486.1, AB089030.1, AB089043.1,  AB089045.1, AB088977.1, AB089028.1, AB088965.1,  AB088951.1, AB089035.1, AB088984.1, AB089032.1 |
| **Lachnospiraceae** | |
| *Butyrivibrio* | U41168.1, X89978.1, AF105403.1, AY178635.1 |
| chicken clone | AF376201.1, AF376218.1 |
| *Clostridium* | AF067965.1, AY169415.1 |
| *Eubacterium* | AB008552.1 |
| *Fusobacterium* | X85022.1 |
| Granular sludge clone | AF332711.1, AF332720.1, AF332721.1 |
| human colonic clone | AJ408957.1, AJ408972.1, AJ408993.1, AJ408989.1 |
| *Lachnospira* | AY169414.1 |
| *Pseudobutyrivibrio* | AF202260.1 |
| Swine intestine clone | AF371584.1, AF371541.1, AF371648.1 |
| Rumen clone | AB034003.1, AB034059.1 |
| Termite gut clone | AB100463.1, AB089002.1, AB088983.2, AB088950.1, B088993.1, AB088990.1, AB088989.1, AB088998.1, AB088994.1, B089044.1, AB088968.1, AB088980.1, AB088952.1, AB089040.1, B088991.1, AB089034.1 |
| **Peptostreptococcaceae** | |
| *Anaerococcus* | AF542229.1 |
| *Finegoldia* | AB109771.1 |
| municipal wastewater treatment plant clone | CR933145.1 |
| oral clone | AF538856.1, Y134903.1, AY134904.1 |
| *Peptostreptococcus* | AF481225.1 |
| TCE-dechlorinating clone | AY217429.1 |
| termite gut clone | AB100461.1, AB062845.1, AB088986.1, AB088954.2, AB088971.1, AB088960.2, AB088970.1 |
| **Prevotellaceae** | |
| Cow rumen clone | AY244919.1, AY244931.1, AY244923.1, AY244900.1 |
| Deep marine sediment clone | AY093462.1 |
| *Prevotella* | AY699286.1, AY134905.1, AF481227.1 |
| Rumen clone | AB185583.1 |
| Swine intestine clone | AF371893.1 |
| **Unclassified** | |
| Arthrobacter | X80744.1 |
| Bacillus | AF454298.1 |
| Beta proteobacterium, uncultured | AY082479 |
| chicken cecum clone, uncultured | AF376430 |
| *Chlorob*i, uncultured | AY118151.1 |
| coal effluent wetland clone | AF523883.1, AF523884.1 |
| cow rumen clone | AY244902.1 |
| *Cryptoendolith* | AY250886.1 |
| DCP-dechlorinating clone | AJ306749.1, AJ306746.2, AJ306774.1, AJ306741.2, AJ306793.1 |
| deep marine sediment clone | AY093480.1 |
| Delaware River estuary clone | AY274839.1 |
| *Desulfotomaculum* | AY084078.1, Y11573.1, Y11574.1 |
| Elbe river clone | AJ401113.1 |
| Feedlot manure, uncultured | AF317384.1 |
| *Flexistipes* | AF481216.1 |
| forest soil clone | AY913277.1 |
| forested wetland clone | AF523973.1, AF524015.1 |
| G+C Gram-positive clone | AF465653.1 |
| *Holophaga* | AJ519640 |
| human mouth clone | AY207065.1, AY134895.1 |
| hydrothermal vent sediment clone | AF420340.1 |
| marine sediment above hydrate ridge clone | AJ535231.1 |
| Mono Lake clone | AF507879.2, AF507860.1, AF507869.2, AF507859.1, AF507862.1, AF507892.1 |
| ocean soil, uncultured | AY181050.1 |
| *Paralvinella* | AJ441239.1, AJ441217.1 |
| penguin droppings sediments clone | AY218551.1 |
| *Pleurotus* | AY838556.1 |
| Rocky Mountain alpine soil clone | AY192275.1 AY192273.1 |
| rumen clone | AB185532.1 |
| sludge clone | AB106352.1, AF234699.1, AF368184.1 |
| soil clone | AJ390463.1 |
| sponge clone | AJ347025.1, AJ347055.1 |
| temperate estuarine mud clone | AY216458.1 |
| termite gut homogenate clone | AB088930.1, AB089097.1, AB089109.1, AB089122.1, AB089008.1 |
| Toolik Lake clone | AF534435.1 |
| trichloroethene-contaminated site clone | AF529128.1 |
| uranium mill tailings waste clone | AJ519669.1, AJ519650.1, AJ296549.1, AJ518784.1, AJ519396.1, AJ536867.1 |
| vadose (subterranean water) clone | AY177763.1 |
| vent worm enrichment clone | AJ431235.1 |
