# Supplementary material for: Insight into the bacterial gut microbiome of the North American moose (Alces alces)
Source: BMC Microbiol. 2012 Sep 19;12:212. doi: 10.1186/1471-2180-12-212 (PMC3585231; doi:10.1186/1471-2180-12-212)
Supplement: Additional file 2 — Table S2. Genus/Identifier and GenBank # of sequences in selected families, found in all colon samples (n = 6), sequences are non-exclusive to the colon. [file 1471-2180-12-212-S2.docx]

**Supplementary Table 2** Genus/Identifier and GenBank # of sequences in selected families, found in all colon samples (n=6), sequences are non-exclusive to the colon.

| **Colon** | |
| --- | --- |
| **Genus or Identifier** | **GenBank ASCN #** |
| **Clostridiaceae** | |
| Chicken intestine clone, uncultured | AF429381.1 |
| Chlorobenzene-degrading clone | AJ488075.1 |
| *Clostridium* | AY007244.1, X76750.1, X71852.1, AB093546.1 |
| Cow rumen clone | AY244908.1 |
| Clostridiales oral clone | AF481208 |
| Equine intestine clone | AJ408137.1 |
| Forested wetland clone, uncultured | AF523923 |
| Granular sludge clone | AY261814.1, AF482434.1 |
| Great Artesian Basin clone | AF407695.1 |
| *Lachnospiraceae* | AF550610.1 |
| Swine intestine clone | AF371796.1, AF371790.1, AF371783.1 |
| Termite gut clone | AB100493.1, AB100478.1, AB088977.1, AB100475.1, AB100479.1, AB089043.1, AB089028.1, AB100486.1, AB088951.1, AB088965.1, AB089045.1, AB089032.1, AB100469.1, AB100483.1, AB089035.1,AB089033.1, AB088984.1, AB100476.1, AB089030.1 |
| **Enterobacteriaceae** | |
| *Alterococcus* | AF075271.2 |
| *Citrobacter* | AF025365.1 |
| Coal effluent wetland clone | AF523903.1 |
| *Enterobacter* | AJ550468.1 |
| *Erwinia* | AF141891.1, AF373202.1 |
| *Escherichia* | NC_000913.2 |
| *Klebsiella* | X93216.1 |
| *Kluyvera* | AJ627202.1 |
| *Opitutus* | AY695840.1 |
| *Pantoea* | AF130912.1, AF373198.1 |
| *Raoultella* | AF181574.1 |
| *Salmonella* | U92194.1 |
| *Serratia* | AF124036.1 |
| Soda lake clone, uncultured | AF507000.1 |
| White-tail deer rumen clone | AF084835.1 |
| Lachnospiraceae | |
| *Butyrivibrio* | U41168.1 |
| *Clostridium ** | AY169415.1 |
| Chicken clone, uncultured | AF376205.1, AF376218.1, AF376201.1 |
| *Fusobacterium* | X85022.1 |
| granular sludge clone | AF332721.1, AF332720.1, AF332711.1 |
| human colonic clone | AJ408972.1, AJ408989.1 |
| *Lachnospira* | AY169414.1 |
| *Roseburia* | AY804149.1 |
| Rumen clone | AB034059.1, AB034003.1 |
| Ruminantium | AB008552.1 |
| Swine intestine clone | AF371584.1, AF371541.1, AF371648.1 |
| Termite gut homogenate clone | AB088950.1, AB089040.1, AB088950.1, AB088990.1, AB088998.1, AB088993.1, AB100463.1, AB088994.1, AB089002.1, AB089000.1, AB089044.1, AB088952.1, AB089036.1, AB089034.1, AB088983.2, AB088980.1, AB088968.1, AB088991.1 |
| **Peptostreptococcaceae** | |
| *Anaerococcus* | AF542229.1 |
| *Finegoldia* | AB109771.1 |
| municipal wastewater treatment plant clone | CR933145.1 |
| Oral clone | AY134904.1 |
| *Peptostreptococcus* | AF481225.1 |
| Swine manure clone | AY167963.1 |
| TCE-dechlorinating clone | AY217429.1 |
| Termite gut clone | AB088971.1, AB062845.1, AB088954.2, AB088986.1, AB088970.1 |
| **Unclassified** | |
| Anaerobic bioreactor clone | AJ278169.1 |
| Antarctic sediment clone | AY250886.1, AY133397.1, AY177804.1 |
| *Arthrobacter* | X80744.1 |
| *Bacillus* | AF454298.1 |
| Bacteroidetes, uncultured | AF449785.1 |
| Beta proteobacterium, uncultured | AY082479 |
| Brackish mud clone, uncultured | AF211303.1 |
| Chicken cecum, uncultured | AF376430 |
| *Chlorobi* clone, uncultured | AY118151.1 |
| cow rumen clone | AY244902.1, AB185532.1 |
| DCP-dechlorinating clone | AJ306793.1, AJ306749.1 |
| Delaware River estuary clone | AY274839.1 |
| *Desulfotomaculum* | Y11574.1, Y11573.1, AY084078.1 |
| Feedlot manure clone, uncultured | AF317384.1 |
| *Ferribacter* | AF282254.1, AF282252.1 |
| Forest soil clone, uncultured | AY913277.1, AF507760.1 |
| forested wetland clone | AF523973.1, AF524015.1 |
| *Holophaga* | AJ519640 |
| hot spring clone | AF027097.1 |
| Human mouth clone, uncultured | AF515500.1, AY207065.1 |
| hydrothermal sediment clone | AF420340.1, U15103.1 |
| Mono Lake clone | AF507859.1, AF507869.2, AF507892.1, AF507879.2 |
| Ocean sediment clone, uncultured | AY181050.1, AY114325.1, AY093473.1 |
| Oral clone | AY134895.1 |
| *Paralvinella* | AJ441239.1 |
| Penguin droppings sediment clone | AY218551.1 |
| *Plectonema* | AF091110.1 |
| Rocky Mountain alpine soil clone | AY192273.1, AY192275.1 |
| *Salmonella* | AF029226.1 |
| sludge clone | AF234699.1 |
| soil clone | AJ390463.1 |
| sponge clone | AJ347055.1 |
| temperate estuarine mud clone | AY216458.1 |
| termite gut clone | AB089097.1, AB089109.1, AB089074.1, AB089008.1 |
| uranium mining waste pile clone | AJ519397.1, AJ518784.1, AJ519663.1, AJ519396.1, AJ532728.1 |
| vent worm enrichment clone | AJ431234.1, AJ431235.1 |

* This entry is included under family Lachnospiraceae in PhyloTrac, and as family Clostridiaceae in GenBank.
